# Identification of potential protein biomarkers for early detection of pregnancy in cow urine using 2D DIGE and label free quantitation

**DOI:** 10.1186/s12014-016-9116-y

**Published:** 2016-07-15

**Authors:** Preeti Rawat, Shveta Bathla, Rubina Baithalu, Munna Lal Yadav, Sudarshan Kumar, Syed Azamal Ali, Anurag Tiwari, Masoud Lotfan, Jasmine Naru, Manoj Jena, Pradip Behere, Ashok K. Balhara, Rajesh Vashisth, Inderjeet Singh, Ajay Dang, Jai K. Kaushik, Tushar K. Mohanty, Ashok K. Mohanty

**Affiliations:** National Dairy Research Institute, Karnal, 132001 India; Central Institute for Research on Buffaloes, Hisar, 125001 India; Bruker Daltonics, Bangalore, India

**Keywords:** Urine, Biomarker, DIGE, LFQ, Mass spectrometry, Gene Ontology, Network construction

## Abstract

**Background:**

An early, reliable and noninvasive method of early pregnancy diagnosis is prerequisite for efficient reproductive management in dairy industry. The early detection of pregnancy also help in to reduce the calving interval and rebreeding time which is beneficial for industries as well as farmers. The aim of this work is to identify potential biomarker for pregnancy detection at earlier stages (16–25 days). To achieve this goal we performed DIGE and LFQ for identification of protein which has significant differential expression during pregnancy.

**Results:**

DIGE experiment revealed a total of eleven differentially expressed proteins out of which nine were up regulated having fold change ≥1.5 in all time points. The LFQ data analysis revealed 195 differentially expressed proteins (DEPs) out of 28 proteins were up-regulated and 40 down regulated having significant fold change ≥1.5 and ≤0.6 respectively. Bioinformatics analysis of DEPs showed that a majority of proteins were involved in regulation of leukocyte immunity, endopeptidase inhibitor activity, regulation of peptidase activity and polysaccharide binding.

**Conclusion:**

This is first report on differentially expressed protein during various time points of pregnancy in cow to our best knowledge. In our work, we identified few proteins such MBP, SERPIN, IGF which were differentially expressed and actively involved in various activities related to pregnancy such as embryo implantation, establishment and maintenance of pregnancy. Due to their involvement in these events, these can be considered as biomarker for pregnancy but further validation of is required.

**Electronic supplementary material:**

The online version of this article (doi:10.1186/s12014-016-9116-y) contains supplementary material, which is available to authorized users.

## Background

An early and precise pregnancy diagnosis is an important criterion for better reproductive management in livestock like cows and buffaloes. Currently different methods (direct and indirect) are in use for diagnosis of pregnancy. The direct methods include per rectal palpation and ultrasonography. However, their application is limited in terms of accurate detection by day 45th and 30th day using per rectal palpation and ultrasonography respectively [1, 2]. Additionally, the expertise of experienced veterinarian is required for confirmed pregnancy diagnosis. The indirect methods include immunological based assay for detection and quantitation of target proteins (Pregnancy Associated Glycoprotein: PAG) and hormones such as progesterone (P_4_), pregnadiol, interferon tau related to pregnancy [3, 4]. However, these methods have inherent limitations of specificity and false positive results in ELISA. Worldwide, different research groups have used urine as a non-invasive source for detection of pregnancy and various other diseases in human being. Pregnancy diagnosis (PD) in dairy animals has remained elusive till date. In fact, dairy animals (cow, buffalo, sheep and goat) although domesticated since time immemorial offer inherent challenges in the understanding of their anatomy, physiology and behavior. Pregnancy in human being is currently detected by presence of human chorionic gonadotropin (HCG) in urine. However, this hormone is absent in bovine urine. Therefore, till date early pregnancy detection in bovine has not been possible [5–7]. After conception, numerous biomolecules such as steroids, prostaglandins and proteins are expressed during early pregnancy [8]. Many of these hormones and proteins are of fetal-placental origin rather than of maternal origin [9]. They are required for the successful establishment of pregnancy and the proliferation of normal and neoplastic cells. Early pregnancy factor (EPF) is one protein which has been observed in the serum of cows during early pregnancy. However, EPF is not confined to pregnancy specifically but also detected in the serum of patients and different animals bearing a variety of tumors [10].

The increased expression of PAG has also been reported in serum and milk during pregnancy in bovine. PAGs are expressed specifically in the maternal and embryonic regions of placenta and belong to aspartic protease family. Different isoforms of PAGs have been reported in bovine during various stages of gestation. The presence of this protein after 28th day post AI serves as an indicator of pregnancy [11]. However, this protein has inherent limitation because its maintenance of basal level of expression till 3 months after parturition. No other proteins till now have been suggested as suitable biomarker for early detection of pregnancy. Thus, although there have been many attempts to develop diagnostics for the detection of early pregnancy in cattle, no success has been achieved till date.

Progress in the field of protein separation and identification technologies has accelerated research into biofluids proteomics for protein biomarker discovery. Urine is considered as an ideal source of biological material for biomarker discovery as it is non-invasive in comparison to other body fluids [12]. Lack of reliable cow-side early pregnancy diagnosis method further aggravates the situation. Urine is an ideal and a rich source of biomarkers in proteomics to analyze the differential expression of urinary proteins in various altered physiological conditions such as pregnancy and different diseases [13] in livestock. The advancement of molecular techniques such as proteomics and their applications in animal research has given a new hope to look for pregnancy biomarkers. In the present investigation, we have identified and analyzed differentially expressed proteins urine of pregnant and non-pregnant cattle on different days of pregnancy using DIGE and Label Free Quantitation (LFQ).

## Methods

### Animal selection and sampling

Karan Fries (KF) heifers from the dairy herd of National Dairy Research Institute, Karnal, India were maintained under expert veterinary supervision. For the present investigation, one litre urine was collected from individual animal (n = 6) in urine bags on different days of pregnancy (0, 16, 22 and 35 days). Day 0 represents the control (collection of urine before artificial insemination: AI). Following AI, urine was collected the cows till the 60th day of pregnancy. Immediately after urine collection, phenylmethylsulfonyl fluoride (PMSF, 0.01 %) was added to prevent proteolytic degradation.

### Confirmation of pregnancy using transrectal-ultrasonography

The transrectal-ultrasonography (Aloka Prosound, Switzerland) was done on the 30th day after breeding and repeated after 45 days post breeding for confirmation. The scanning of the uterus and ovaries was done using a 6.5 MHz rectal linear probe (Aloka UST-5820-5, Switzerland). Pregnancy diagnosis was confirmed by observation of embryocoele and allantoic fluid [14]. The ovaries were scanned for the presence of corpus luteum also.

### Sample preparation

Insoluble material in urine was removed by centrifugation at 6000 rpm for 30 min, followed by diafiltration with phosphate buffer saline (PBS, pH 7.5) (133 mM NaCl, 2.7 mM KCl, 10 mM Na_2_HPO_4_ and 2 mM KH_2_PO_4_) [12, 15]. The diafiltered urine was concentrated up to 100 ml by using 3 kDa hollow fibre cartridge in Marlow Benchtop System (GE healthcare, USA). Protease inhibitor cocktail (Sigma, USA) was added to the concentrated urine to prevent proteolysis and stored at −80 °C till further use.

### Protein precipitation

Protein precipitation from the concentrated urine was performed by Proteo Spin Maxi Kit (Norgen Biotek, USA) following manufacturer’s instructions. Briefly, the pH of the urine sample was adjusted to 3.5 by adding a binding buffer. The Proteo Spin column was activated by adding 5 ml of the column activation and wash buffer and centrifuged for 3 min at 1000×*g*. The flow through was discarded and same step was repeated twice and 20 ml of the pH adjusted urine was loaded onto the column and centrifuged for 5 min at 1000×*g*. The column was again washed by applying column activation and wash buffer and centrifuged for 3 min at 1000×*g*. Protein was eluted with elution buffer (10 mM Na_2_HPO_4_, pH 12.5) in a fresh collection tube containing the neutralizer. The eluted proteins were concentrated and preserved at −80 °C until further analysis [16].

### Clean up

Interfering substances such as salts, detergents, nucleic acid, etc. were removed from the precipitated urinary proteins using 2-D clean-Up kit (GE healthcare, USA) and the resulting pellet were rehydrated in lysis buffer (7 M Urea, 2 M Thiourea, 4 % CHAPS, 30 mM Tris). Protein concentration was estimated using 2-D Quant kit (GE Healthcare, USA) as per manufacturer’s instructions with bovine serum albumin as a standard.

## 1D SDS-PAGE

The individual proteins were precipitated and analysed by (10 × 10.5 cm) SDS-PAGE with 4 % stacking and 12 % resolving gel using MiniVE gel electrophoresis apparatus (GE healthcare, USA). The gels were stained with Coomassie Brilliant Blue G 250 (Bio-Rad Laboratories, USA) for 1 h and destained.

### Sample labelling with fluorescent dyes

The sample pH was adjusted to 8.5 by 100 mM NaOH. An equal amount of proteins was pooled (n = 6) separately to make a final amount of 15 µg for each day of sample i.e. 0, 16, 22 and 35 days, the protein samples were labelled with 200 pmol Cy3 (non-pregnant) and Cy5 (pregnant) respectively. Internal standard (pooled sample, 7.5 µg each) was labelled with 200 pmol Cy2 dye. Dye swapping was done to avoid dye biasness by labelling with 200 pmol Cy5 (non-pregnant) and Cy3 (pregnant) respectively. The whole labelling procedure was performed on ice, after labelling samples are incubated in dark for 30 min. Subsequently, 1 µl of 10 mM lysine was added to quench the reaction. The samples were incubated for 10 min on ice in dark and mixed as per the experimental design (Table 1). The final sample volume was made 125 µl for each strip, by adding De Streak rehydration buffer (GE Healthcare). Six IPG (7 cm, pH 4–7, GE Healthcare) were rehydrated by passive rehydration with labelled sample for 16 h at room temperature following the protocol described by Jena et al [17].

**Table 1:** Experimental design for DIGE experiment (0, 16, 22 and 35 day of pregnancy)

Gels	Cy2	Cy3	Cy5
Gel 1	15 μg (3.75 μg each of samples 0,16, 22 and 35 days)	15 μg sample 0 day	15 μg sample 16 day
Gel 2	15 μg (3.75 μg each of samples 0,16, 22 and 35 days)	15 μg sample 0 day	15 μg sample 22 day
Gel 3	15 μg (3.75 μg each of samples 0,16, 22 and 35 days)	15 μg sample 0 day	15 μg sample 35 day
Gel 4	15 μg (3.75 μg each of samples 0,16, 22 and 35 days)	15 μg sample 16 day	15 μg sample 0 day
Gel 5	15 μg (3.75 μg each of samples 0,16, 22 and 35 days)	15 μg sample 22 day	15 μg sample 0 day
Gel 6	15 μg (3.75 μg each of samples 0,16, 22 and 35 days)	15 μg sample 35 day	15 μg sample 0 day

## 2D GE and image scanning

Isoelectric focusing (IEF) was performed with the parameters 150 V for 1 h 20 min (step), 300 V for 20 min (grad), 5000 V for 1 h 40 min (grad), 5000 V for 25 min (step) with a total of 7000 Vh. Thereafter, strips were equilibrated with an equilibration buffer (6 M Urea, 50 mM Tris pH 8.8, 2 % SDS, 30 % Glycerol and 0.02 % Bromophenol Blue) containing 1 % DTT for 15 min (reduction) and followed by an equilibration buffer containing 2.5 % iodoacetamide for another 15 min (alkylation). SDS-PAGE of 6 gels was performed in MiniVE (GE healthcare, USA) electrophoresis system (10 × 10.5 cm) with 12 % resolving gel. After electrophoresis, gels were scanned with typhoon Trio+ variable mode imager (GE Healthcare) by using the parameters followed earlier with minor modifications [17, 18]. Briefly, the gels were scanned with 100 µm resolution and normal sensitivity. Cy2 images were scanned with 575 nm (blue) laser and 520 BP40 emission filter, Cy3 images were scanned with 515 nm (green) laser and 580 BP30 emission filter and Cy5 images were scanned with 490 nm (red) laser and 670 BP30 emission filter.

### Image analysis and spot picking

Scanned images were analyzed in the Decyder 2-D software (version 7.0, GE Healthcare) to identify expression of proteins. Estimated number of spots was set to 2000 and in individual gel spots were detected by Differential In-Gel analysis (DIA). All images from 6 different gels were matched through Biological Variation Analysis (BVA) which provides statistical data for differentially expressed proteins (above 1.5 fold, p ≤ 0.05) between three experimental groups. A total of 11 differentially expressed protein spots were identified.

### Preparative gel and spot digestion

A preparative gel having 320 µg pooled (n = 6) proteins from various days of pregnant animals (0, 16, 22 and 35 days) was carried out using the same parameters used for DIGE as mentioned above and stained with Coomassie Brilliant Blue (R-350) followed by destaining. Selected spots were picked from preparative gel and transferred into 1.5 ml Eppendorf tubes, spots were washed with Milli-Q water and 40 mM NH_4_HCO_3_ in 50 % ACN (1:1) and for rehydration 100 µl of 100 % ACN were added to each tube and incubated for 10 min, ACN was carefully discarded and for reduction 10 mM DTT in 40 mM NH_4_HCO_3_ buffer was added and incubated for 15 min, then alkylation was done in 55 mM iodoacetamide in 40 mM NH_4_HCO_3_ buffer. Spots were washed and rehydrated. For tryptic digestion spots were covered with trypsin solution (12.5 ng/µl in 50 mM NH_4_HCO_3_) for 45 min in ice. Trypsin digestion was performed overnight at 37 °C and stopped by adding 5 % formic acid. The extracted peptides were dried in a Speed-Vac and desalted by using Ziptip (Millipore, USA) and identified by Nano-LC-MS/MS.

### In- solution digestion

For in-solution digestion, 20 μg of pooled samples (n = 6) from non-pregnant and pregnant cows (0, 16, 22 and 35 days) were collected on different days of pregnancy was processed. In solution digestion method was performed as reported earlier with slight modification [16]. In brief, 45 mM DTT in 50 mM NH_4_HCO_3_ was used to reduce disulfide bonds followed by alkylation of cysteine residues using 10 mM IAA in 50 mM NH_4_HCO_3_. Digestion was carried out overnight using trypsin (1:20) (modified sequencing grade; Promega, USA) at 37 °C. The reaction was subsequently stopped with 10 % TFA, peptides were vacuum dried, desalted by zip tip and stored at −80 °C.

### LC-MS/MS and data analysis for label free quantitation (LFQ)

The digested peptides were reconstituted in 0.1 % formic acid in LC/MS grade water and subjected to nano-LC (Nano-Advance, Bruker, Germany) followed by identification in captive spray-Maxis-HD qTOF (Bruker, Germany) mass spectrometer (MS) with high mass accuracy and sensitivity. The peptides were enriched by nano trap column (Bruker Magic C_18_AQ, particle size-5 μm, pore size-200 Å) and separated on an analytical column (Bruker Magic C_18_AQ, 0.1 × 150 mm, 3 μm particle size, and 200 Å pore size) at flow rate 800 nl/min and eluted using a linear gradient of 5−45 % acetonitrile over 135 min. The MS/MS scan was carried out at m/z range of 400–1400 followed in data dependant mode. For each cycle, the six most intense precursor ions from survey scan were selected for MS/MS [16]. The identification and quantitation was done using MS/MS spectra.

### Data processing and bioinformatics analysis

MS data were analyzed using MaxQuant [19] software version 1.5.0.8 and searched with UniProt *Bos taurus* and *Bubalus bubalis* database along with common contamination sequences. Database search was performed in MaxQuant environment integrated with Andromeda. For searching, the enzyme specificity was set to trypsin with the maximum number of two missed cleavages. The precursor mass tolerance was assigned to 0.07 Da for the first search and 0.006 Da for the main search. Mass tolerance for matching peaks to theoretical ion series was set to 40 ppm. The false discovery rate (FDR) for PSM, protein, and site decoy fraction was set to 1 %. The search included variable modifications of protein N-terminal acetylation, methionine oxidation, and carbamidomethylation of cysteines was searched as a fixed modification. The maximal number of modifications per peptide was set to be 6. The minimum peptide length of 6 was set, and the ‘peptide re-quantification’ function was enabled. To validate and transfer identifications across different runs, the ‘match between runs’ option in MaxQuant was enabled with a retention time window of 0.7 min and an alignment time window of 20 min. Subsequent bioinformatics analysis were performed using Protein Analysis Through Evolutionary Relationships (PANTHER) to compare the GOBP, GOCC, GOMF and GOPC. The obtained PANTHER [20] data was further analyzed and graphs were prepared using MS Excel 2007. The mass spectrometry proteomics data have been deposited to the ProteomeXchange consortium with the PRIDE partner repository with the database identifier PXD004122.

## Result and discussion

**Fig. 1:**
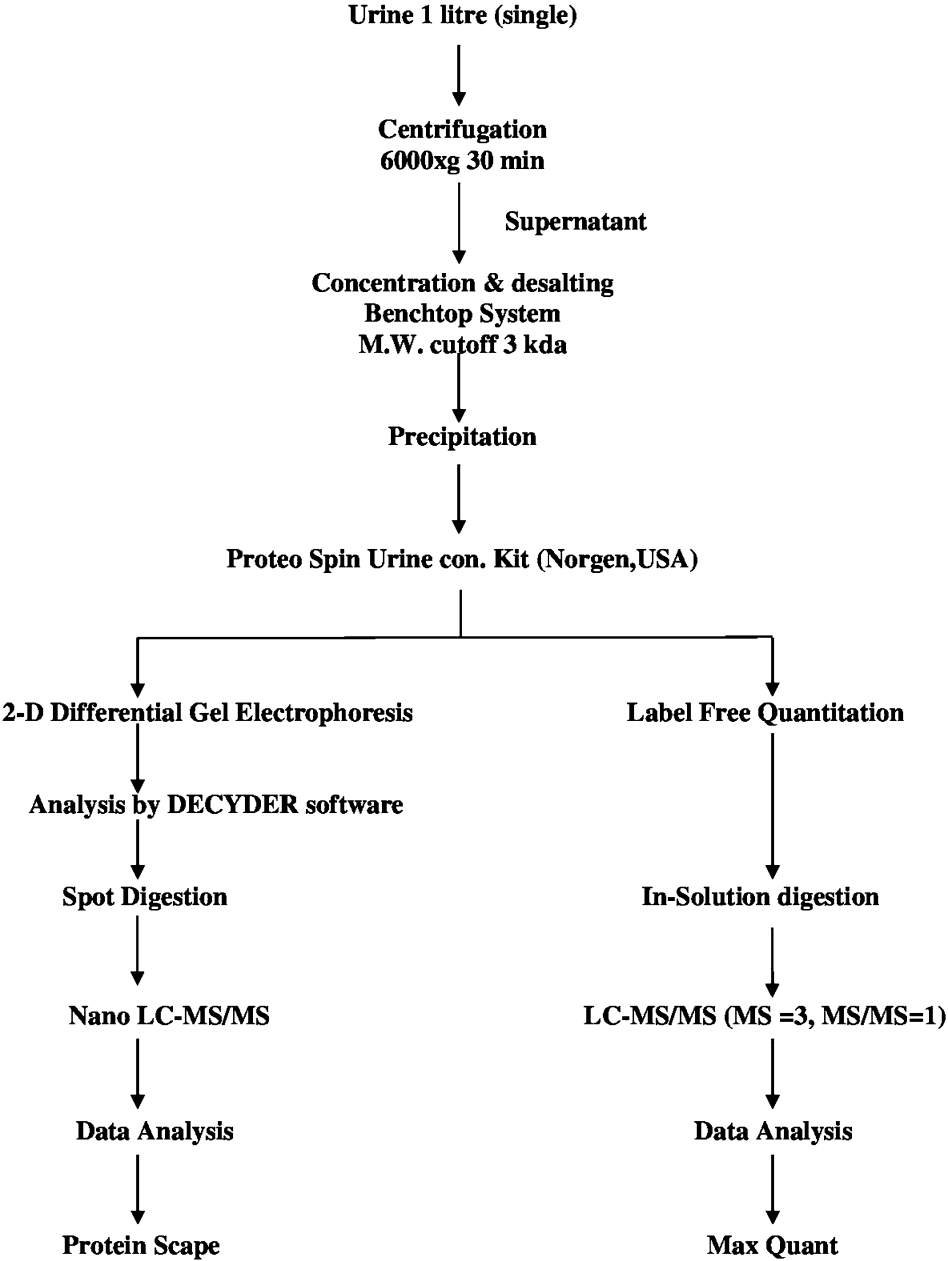
Workflow of the DIGE and LFQ for identification of differential expressed proteins during various time points of pregnancy

Urine is considered to be the best source of biological material for diagnosis of altered physiological and various patho-physiological conditions due to its non-invasive nature and collection in large volume [12]. It is a well known fact that pregnancy affects the protein expression in maternal serum and urine. Furthermore, the quantitative difference in protein expression during pregnancy is useful for the detection of biomarkers related to pregnancy. In the present investigation, we have used gel based (DIGE) and non-gel based approaches (LFQ) to identify differentially expressed proteins during early pregnancy in cattle (Fig. 1). The present study aimed to identify protein biomarkers which can possibly be used for detection of pregnancy at an earlier stage (16–25 days) in cow urine samples which will be beneficial for dairy farmers.

### Identification of differentially expressed proteins (DEP) using DIGE

**Fig. 2:**
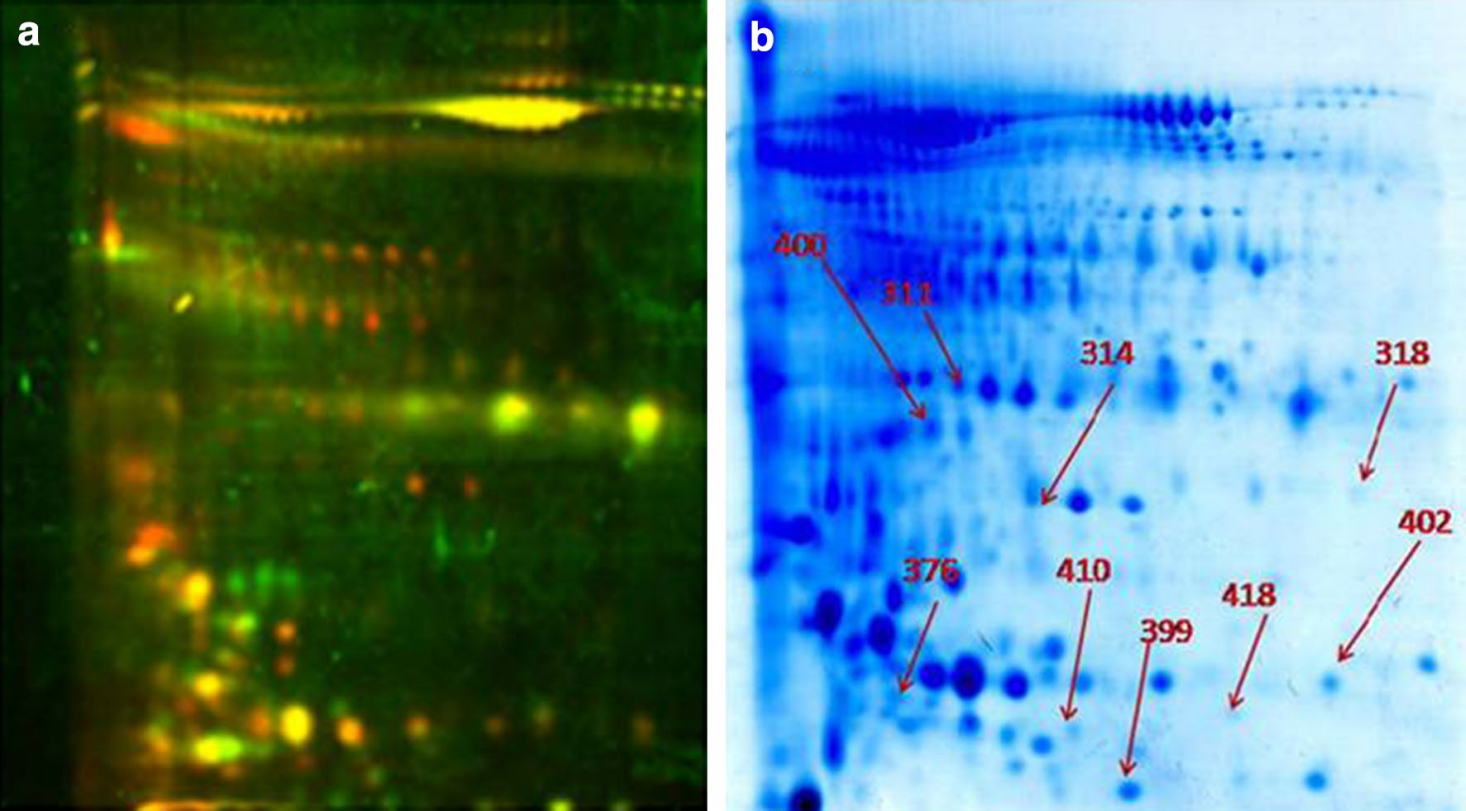
**a** Images of DIGE gels scanned using Typhoon Scanner. **b** Image of preparative gel (320 µg protein on 7 cm IPG strip having pI-4-7 and 12 % separating gel) used for picking differential expressed proteins

We used DIGE approach to identify the differentially expressed proteins during different days of pregnancy, such as days 0 (non-pregnant control), 16, 22 and 35 post breeding. A representative image of the DIGE gel in the present investigation is shown in Fig. 2a, b. Additional figures of all DIGE gels are shown in Additional file 1: Figure S1. After analysis of the DIGE gel in Decyder software, we observed a total of 11 differentially expressed proteins (DEPs) having fold change of ±1.5 (*p* ≤ 0.05). Out of 11 DEPs, 9 proteins were up-regulated (Table 2). We have discussed functional relevance of few selected proteins namely Alpha 2HS Glycoprotein (A2HS), AMBP, Renin, Mannan-binding protein which may have role in pregnancy associated events. Alpha-2-HS (Heremans-Schmid) glycoprotein also known as Fetuin-A is a phosphoprotein which is mainly expressed in the liver, the tongue, and placenta in humans [21]. It is expressed in higher concentrations in serum and amniotic fluid during fetal life and is also involved in development-associated regulation of calcium metabolism and osteogenesis. The increased expression of this protein has been reported during pregnancy in women [13]. Interestingly, we observed secretion of this protein in the urine of pregnant cows during early pregnancy. The renin–angiotensin system (RAS) is mainly associated with the regulation of blood pressure and ion homeostasis. Angiotensin II (Ang II) which is generated due to the proteolytic action of rennin has been reported to influence oviductal gamete movements and foetal development. The pre-implanted embryo responds to Ang II from mothers rather than from embryos. It has been suggested that maternal RAS influences blastocyst hatching and early embryonic development [22]. Alpha-2 Macroglobulin (AMBP) is a protease inhibitor and has been reported to prevent excessive trophoblastic invasion. AMBP reportedly influences trophoblast invasion in human pregnancy, which would be reflected in its increased production in the decidua basalis [23]. We also observed up-regulation of Mannan-binding protein (MBP) in our experiment. MBP is a mannan-binding lectin which is secreted into the amniotic fluid and its functional activity is mediated through the formation of mannose-binding lectin and mannose-binding lectin-associated serine protease 2 complexes (MBL-MASP2 complex). This complex is actively involved in mannose-binding lectin complement pathway resulting in antibody-independent recognition and clearance of pathogen in the amniotic cavity during pregnancy [24, 25]. Increased secretion of MBP in urine during early pregnancy suggests its possible application as a potential biomarker.

**Table 2:** List of differentially expressed proteins

Spot ID	Protein	Gene symbol	16 vs 0	22 vs 0	35 vs 0	Score	Seq. coverage (%)	MW (kDa)	pI	No. of peptides
311	Alpha-1B-glycoprotein	AIBG	−1.0	−1.2	1.5	112.75	5.00	53.50	5.30	2
318	Protein AMBP	AMBP	1.8	2.0	1.2	425.61	22.70	39.20	7.81	8
376	Allergen Bos d 2	ALL2	2.3	−1.0	1.1	654.31	40.70	19.50	4.66	10
399	Transthyretin	TTHY	1.3	1.5	−1.1	227.61	30.60	15.70	5.90	4
402	Carbonic anhydrase 2	CAH2	1.3	1.7	−1.1	297.08	28.50	29.10	6.41	5
410	Chain L, crystal structure of bovine antibody)		3.2	−1.0	−1.0	59.90	7.90	22.50	5.87	1
418	Cathelicidin-4 precursor		2.0	−1.0	−1.2	458.27	38.20	16.50	6.29	6
314	Renin receptor	RENR	−1.4	−1.4	1.6	268.80	10.80	39.50	5.35	5
400	Mannan-binding lectin serine peptidase 2		1.7	2.1	2.6	103	8.31	50.444	5.37	2

### Identification of differentially expressed proteins by LFQ

**Fig. 3:**
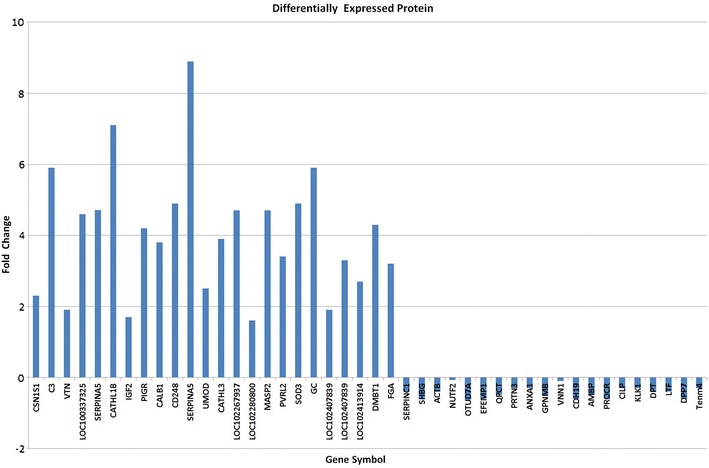
Bar graph of differentially expressed proteins having fold change ≥1.5 and ≤0.6 revealed by Max quant Software for LFQ data

Analysis of the LFQ results using Maxquant software revealed 195 (Additional file 2: Table S1) differentially expressed proteins, out of which 28 proteins were up-regulated and 40 proteins were down-regulated having fold change ≥1.5 and ≤0.6 respectively which were considered for further analysis (Tables 3, 4; Fig. 3). The analysis revealed some important proteins which play a role in pregnancy-associated events such as embryo implantation, establishment, and maintenance of pregnancy. Expression of important proteins such as Hormone-binding globulin, Haptoglobin, SerpinB_3_ like, Uromodulin, Cathelicidin, Mannan-binding protein, uteroglobin, Vitamin-binding protein and Insulin-like growth factor-binding protein II (IGFBP-II) increased significantly during the early days of pregnancy (16–22 days). Uterine serpins are produced by uterine endometrium and regulate the immune function or participate in the trans-placental transport. Expression of Serpin was decreased on day 10 but subsequently increased on day 16 [26]. Another study revealed that there is increased expression of serpin in the endometrium of pregnant cows compared to cyclic heifers during pregnancy recognition period (16–18 days) [27].

**Table 3:** LFQ (max quant) list of up regulated proteins

S. no.	Protein ID	Protein name	Peptides	Sequence coverage (%)	Mol. weight (kDa)	Fold change		
						16 vs 0 days	22 vs 0 days	35 vs 0 days
1	gi|528953246	Alpha-S1-casein isoform X2	2	12.4	20.1	1.5	1.9	1.6
2	gi|741932316	Complement C3 isoform X1	14	11.1	187.2	1.6	5.9	7.0
3	gi|440895896	Vitronectin	8	15.5	53.6	1.7	1.9	2.8
4	gi|741962549	Pigment epithelium-derived factor	2	14	24.2	1.6	4.6	5.0
5	gi|28603766	Plasma serine protease inhibitor precursor	10	31.7	45.2	1.6	4.71	8.1
6	gi|667665642	Cathelicidin, partial	9	57.4	17.6	1.7	7.1	5.1
7	gi|68165959	Insulin-like growth factor II, partial	4	17.6	5.7	1.6	1.7	11.7
8	gi|664764564	Polymeric immunoglobulin receptor	2	1.3	82.9	2.1	4.2	7.1
9	gi|803268763	Calbindin	2	9.2	29.9	1.5	3.8	14.5
10	gi|741969869	Fibulin-2 isoform X1	3	3.9	124.0	1.8	12.7	16.8
11	gi|741905201	Endosialin isoform X1, partial	8	14.9	70.8	4.7	4.9	5.5
12	gi|803331888	Plasma serine protease inhibitor	6	19.3	45.4	1.6	8.9	12.6
13	gi|426254387	Uromodulin	13	19.8	69.8	1.9	2.5	2.5
14	gi|440891627	Cathelicidin-3, partial	2	16.3	15.2	4.7	3.9	5.8
15	gi|440893899	Hypothetical protein M91_15215, partial	2	3.1	69.0	2.2	4.7	8.3
16	gi|440901185	Ig lambda-1 chain C regions, partial	5	68.6	11.1	1.7	1.6	6.1
17	gi|741954224	Mannan-binding lectin serine protease 2 isoform X2	6	34.1	20.3	3.2	4.7	11.8
18	gi|803247194	Nectin-2 isoform X4	3	8.8	51.3	2.4	3.4	9.9
19	gi|528952645	Extracellular superoxide dismutase [Cu-Zn] isoform X1	4	22	26.1	2.1	4.9	10.6
20	gi|528953415	Vitamin D-binding protein isoform X1	6	18.1	53.3	2.8	5.9	4.1
21	gi|440906200	Hypothetical protein M91_02830, partial	2	9.4	19.4	4.3	1.9	1.8
22	gi|594059057	Zinc-alpha-2-glycoprotein-like	2	5.4	34.0	3.0	3.3	2.1
23	gi|594116459	Retinol-binding protein 4-like, partial	2	13	18.5	1.5	2.7	9.5
24	gi|741973898	Zymogen granule protein 16 homolog B-like	3	18.1	16.9	6.2	4.8	1.6
25	gi|741976749	Deleted in malignant brain tumors 1 protein isoform X49	6	13	166.5	7.4	4.3	4.9
26	gi|6137530	B chain B, The crystal structure of calcium-free equine plasma gelsolin	2	3.8	80.5	1.6	6.3	15.8
27	P02672	Fibrinogen alpha chain precursor	3	6.2	67.0	2.1	3.2	3.3
28	gi|741959425	Major allergen I polypeptide chain 2-like	2	21.4	12.6	1.4	3.0	4.3

**Table 4:** List of down regulated proteins

S. no.	Protein ID	Protein name	Peptides	Sequence coverage (%)	Mol. weight (kDa)	Fold change		
						16 vs 0 days	22 vs 0 days	35 vs 0 days
1	gi|594080166	Antithrombin-III	3	1.9	52.4	0.3	0.4	0.5
2	gi|741962906	Sex hormone-binding globulin isoform X2	11	44.8	37.3	0.2	0.6	0.5
3	gi|358420932	Serpin B4-like	8	21.7	44.3	0.1	0.0	0.0
4	gi|57619329	Actin, cytoplasmic 1	9	33.9	41.7	0.1	0.4	0.2
5	gi|803211084	Nuclear transport factor 2	3	45.7	14.4	0.07	0.07	0.7
6	gi|371491843	Peptidoglycal recognition protein 1	6	40	21	0.03	0.0	0.0
7	gi|239919016	Immunoglobulin G3 heavy chain constant region, partial	2	7.3	25	0.34	0.1	4.3
8	gi|109030	Ig lambda chain C region - (fragment)	2	18.1	11.311	0.03695	0.29332	0.04130
9	gi|927774498	A Chain A, Bovine Allergen Bos D 2 In The Monoclinic Space Group C2	7	44.2	17.8	0.5	0.09	0.0
10	gi|194206368	OTU domain-containing protein 7A	2	1	79.606	0.56012	0.63301	0.60267
11	gi|664707994	EGF-containing fibulin-like extracellular matrix protein 1 isoform X1	11	30.1	54.8	0.2	0.6	0.4
12	gi|803030626	Glutaminyl-peptide cyclotransferase isoform X1	8	37.7	41	0.3	0.5	0.5
13	gi|594074072	Ligand isoform X3	2	3.6	0.4	0.6	0.4	0.4
14	gi|803074446	Myeloblastin	2	4.5	24.258	0.10620	0.30618	0.18733
15	gi|803156785	Signal peptide, CUB and EGF-like domain-containing protein 2 isoform X4	2	2	84.1	0.6	0.5	0.2
16	gi|741886482	Neutrophil gelatinase-associated lipocalin	7	42.7	22.8	0.1	0.01	0.02
17	gi|803333753	Annexin A1	12	39.9	38.8	0.2	0.3	0.2
18	gi|803284484	Transmembrane glycoprotein NMB	4	6.5	61.6	0.1	0.6	0.5
19	gi|594070987	Pantetheinase isoform X6	5	17	49.9	0.08	0.1	0.3
20	gi|803234439	Alpha/beta hydrolase domain-containing protein 14B	3	17.1	22.4	0.09	0.5	0.3
21	gi|803299355	Cadherin-19 isoform X1	2	2.6	86.8	0.5	0.6	0.3
22	gi|440901131	Protein AMBP, partial	6	13.5	37.2	0.4	0.5	0.5
23	gi|803219892	Endothelial protein C receptor isoform X4	5	28.6	24.7	0.3	0.6	0.5
24	gi|594051858	Cartilage intermediate layer protein 1 isoform X8	6	6.2	123.9	0.0	0.3	0.1
25	gi|594114749	Kallikrein-1	5	42.9	28.7	0.1	0.3	0.4
26	gi|440904881	Dermatopontin	8	51.3	23.7	0.1	0.4	0.3
27	gi|440905390	Haptoglobin, partial	6	18.5	44.7	0.02	0.05	0.02
28	gi|440909177	Cubilin, partial	12	4.2	396.7	0.01	0.1	0.01
29	gi|594044657	Resistin	2	12.8	11.4	0.1	0.09	0.2
30	gi|440913634	Leukocyte elastase inhibitor	4	11.6	45.1	0.05	0.08	0.03
31	gi|594086536	Collagen alpha-3(VI) chain isoform X4	6	2.8	325.1	0.1	0.4	0.5
32	gi|741942660	EGF-containing fibulin-like extracellular matrix protein 1 isoform X1	14	35.9	54.8	0.2	0.4	0.5
33	gi|528978722	Factor XIIa inhibitor isoform X1	7	20.5	51.7	0.02	0.3	0.2
34	gi|529002050	Lactotransferrin isoform X1	25	37.9	78.0	0.1	0.4	0.05
35	gi|664770863	Dipeptidyl peptidase 2	2	4.9	54.5	0.2	0.6	0.2
36	Q3SZV7	Q3SZV7 Similar to hemopexin	4	14.8	52.2	0.06	0.4	0.1
37	gi|594103691	Allergen Bos d 2-like	2	11.1	18.9	0.1	0.1	0.1
38	gi|741973323	Serpin B3-like isoform X2	5	11.5	48.1	0.08	0.1	0.2
39	gi|440895590	Growth arrest-specific protein 7, partial	2	0	35.4	0.03	0.15	0.1
40	gi|545223253	Teneurin-4	2	0	277.6	0.2	0.3	0.3

The success of pregnancy is dependent on the uterine environment which is mediated by different hormones and growth regulators. Insulin-like growth factors are expressed in embryo and reproductive tract of cow and sheep. They are reportedly involved in blastocyst formation, implantation and embryo growth [28, 29]. We observed up-regulation of IGFBP-II during early pregnancy. IGFBPs bind IGFs with high affinity, regulating the availability of free IGFs. Higher expression of IGFBP-II during early pregnancy suggests that it binds to IGF-II for its optimal bioavailability to embryos during implantation and embryo growth. Haptoglobin is a glycoprotein expressed in uterine epithelium during implantation period [30]. We observed increased expression of this protein during early pregnancy the present study. We also observed increased expression of the vitamin D-binding protein in the urine during early pregnancy. Vitamin D-binding protein belongs to albumin family of proteins and is present in plasma, cerebrospinal and ascitic fluids and on cell surface of many cell types. This protein binds to various plasma metabolites and transport to their targeted sites. Higher expression of Vitamin D-binding protein has been reported in uterus and placenta of bovine during pregnancy [31]. It has been reported that vitamin D-binding protein is also involved in active transport of Ca^+^ which is crucial for the fetal developmental events such as bone mineralization, neuro muscular activities and blood coagulation. The up-regulation of Vitamin D-binding protein in urine during early pregnancy suggests its potential as a biomarker for early detection of pregnancy in cattle. We also observed up regulation of MBP which correlates well with our DIGE data. Expression of uromodulin was also upregulated during early pregnancy in urine which is in agreement with the observation reported earlier [32]. We also identified many proteins during early pregnancy (Table 3) which may be playing important role in pregnancy associated events such as transfer of embryo from fallopian tube, hatching of blastocyst, maintenance and implantation of embryo and fetal development.

### Functional classification of protein

**Fig. 4:**
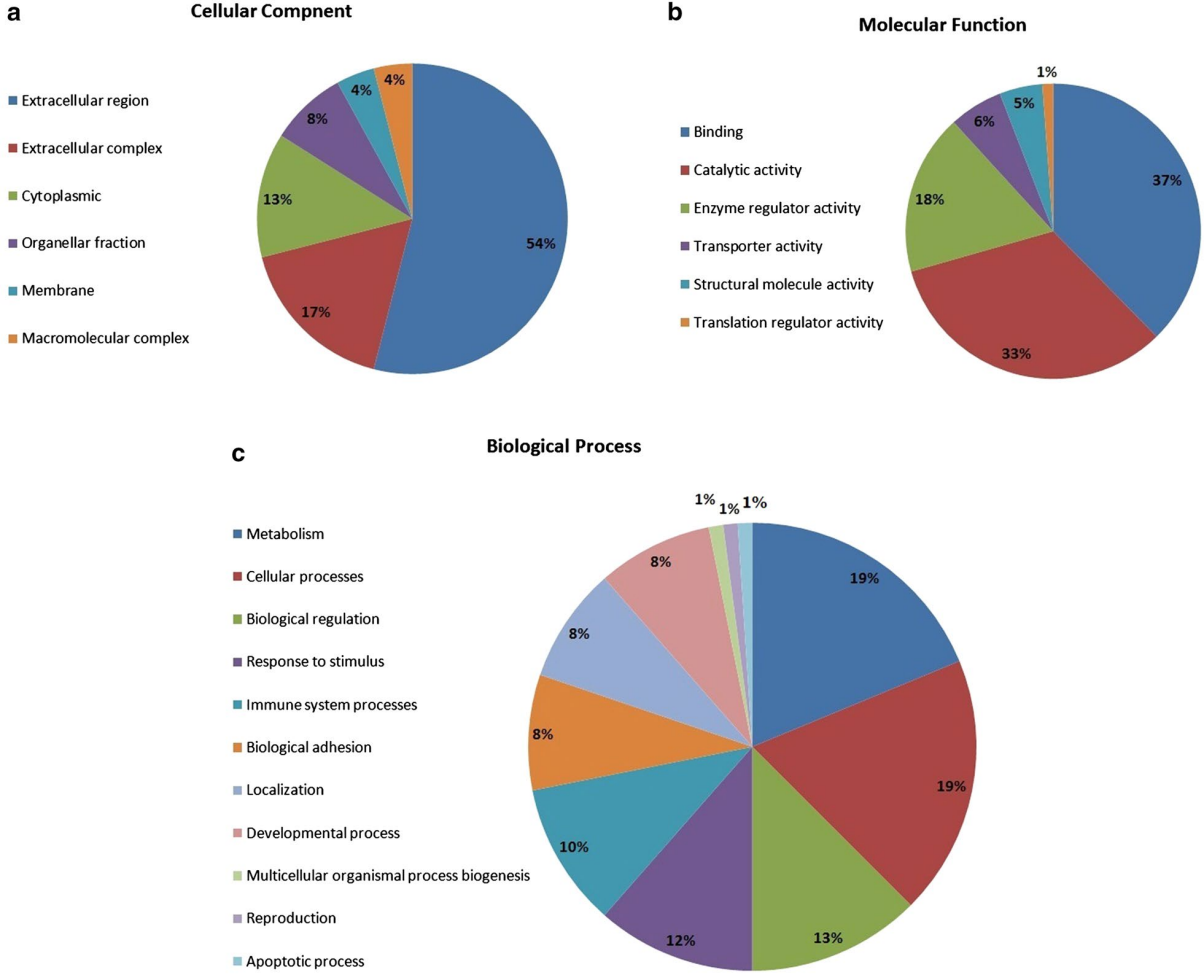
Gene Ontology classification of proteins on the basis of their involvement in **a** cellular component, **b** molecular function, **c** biological process using PANTHER 7.0 software

The functional characterization of identified proteins (195 proteins) was based on Gene Ontology (GO) using PANTHER 8.0 bioinformatics software platform, which generated information regarding cellular localization, metabolic and biological process. The classification based on cellular component (Fig. 4a) revealed that the majority of proteins are present at extracellular region (54 %), followed by extracellular complex (17 %), cytoplasmic (13 %), organellar fraction (8 %), membrane and macromolecular complex (4 %). Classification based on molecular function (Fig. 4b) showed that a large majority of the proteins are involved in binding (32 %), catalytic activity (28 %), enzyme regulator activity (15 %), transporter activity (5 %), structural molecule activity (4 %) and translation regulator activity (1 %). On the basis of biological processes (Fig. 4c), the proteins were classified into those involved in metabolism and cellular processes (18 %), biological regulation (12 %), response to stimulus (11 %), immune system processes (10 %), biological adhesion, localization and developmental process (8 %), multicellular organismal process biogenesis, reproduction and apoptotic process (1 %).

### Network generation and visualization

**Fig. 5:**
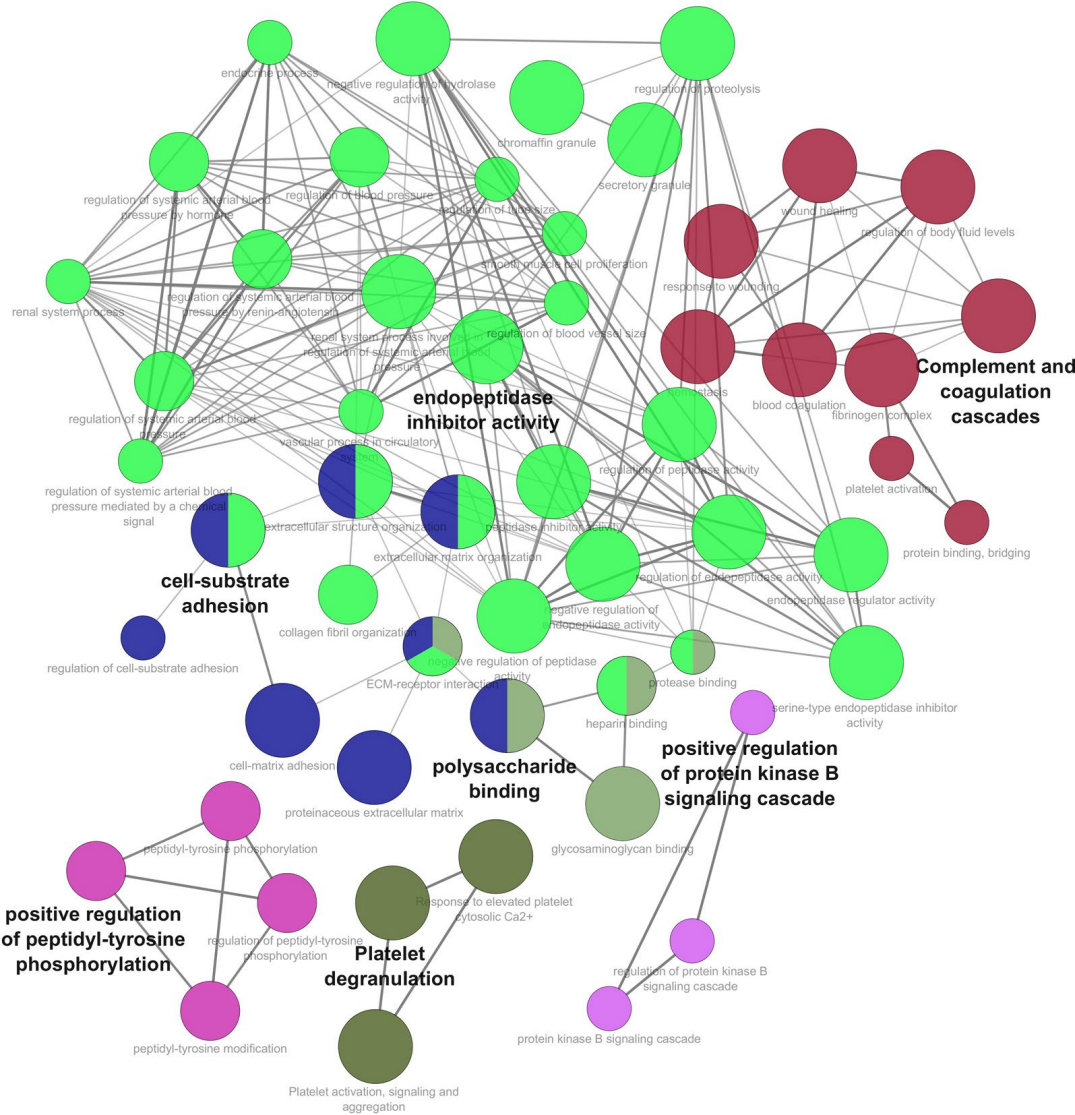
Network construction for protein–protein interaction study was done using Cytoscape software with ClueGO plug-in

To create protein–protein interaction network for identified urinary proteins, offline software tool Cytoscape was used along with plug-in ClueGO. The annotations network of ClueGO provides the biological significance of identified differentially expressed 195 bovine urinary proteins. ClueGO initially generates a binary gene-term matrix with the particular terms and their associated partner genes. The generated network shows the proteins as nodes that are linked through edges. During the search, the majority of the proteins were clustered into pathways (Fig. 5). From these results, four discrete pathways were recognized comprising regulation often do peptidase inhibitor activity, complement coagulation cascades, polysaccharide-binding positive regulation of peptidyl-tyrosine phosphorylation and protein kinase B signalling cascade. Regulation of these events is associated with various immunological functions. This protects the system from systemic infection and employs a number of strategies for the recognition and clearance by the host immune system [33]. Pregnancy is an event when a foreign body starts growing in the womb of pregnant mother and the system reacts to the foreign body by activation of complement C pathway and induction of endopeptidases. Concomitantly, a set of endogenous protease inhibitors are also expressed in the system which may possibly protect the embryo and young foetus from the proteolytic assault and immune rejection. A large number of peptidase inhibitors e.g. AGT, AHSG, AMBP, C3, COL6A3, GAS6, KNG1, LOC784932, PAPLN, SERPINA1, SERPINF2 were identified which are involved in controlling the activity of various serine and cysteine-type endopeptidase. These protease inhibitors possibly maintains the immune system from proteolytic insult.

## Conclusion

 Although we have identified a good number of differentially expressed proteins, further validation is required to authenticate their suitability as potential biomarkers for early detection of pregnancy. The validation with advancement in high throughput mass spectrometry targeted proteomics approach is an ideal method to validate these potential biomarkers which will be part of another study. To the best of our knowledge, the present investigation reports gel based (DIGE) and non-gel based (LFQ) differential proteome profiling in pregnant *vis*-*a*-*vis* non-pregnant Karan Fries cows for the first time. It provides us important information on differentially expressed urinary proteins during early pregnancy which possibly encourages the research community and dairy industry for development of urine based pregnancy diagnostic assay for early detection of pregnancy in cattle.

## Additional files

10.1186/s12014-016-9116-y Supplimentary Table: Total identified proteins revelaed by Max quant Software.
10.1186/s12014-016-9116-y List of identified proteins revealed by Max Quant Softawre.

## Abbreviations

DIGE: difference gel electrophoresis
MS: mass Spectrometry
LFQ: label free quantitation
DEP: differentially expressed proteins
MBP: mannan binding protein
IGF: insulin like growth factor
PAG: pregnancy associated glycoprotein
HCG: human chorionic gonadotropin
PD: pregnancy diagnosis
EPF: early pregnancy factor
AI: artificial insemination
DIA: differential In-Gel analysis
BVA: biological Variation Analysis
GO: gene Ontology
