# Supplementary figures and images for: Identification of potential protein biomarkers for early detection of pregnancy in cow urine using 2D DIGE and label free quantitation
Source: Clin Proteomics. 2016 Jul 15;13:15. doi: 10.1186/s12014-016-9116-y (PMC4946208; doi:10.1186/s12014-016-9116-y)

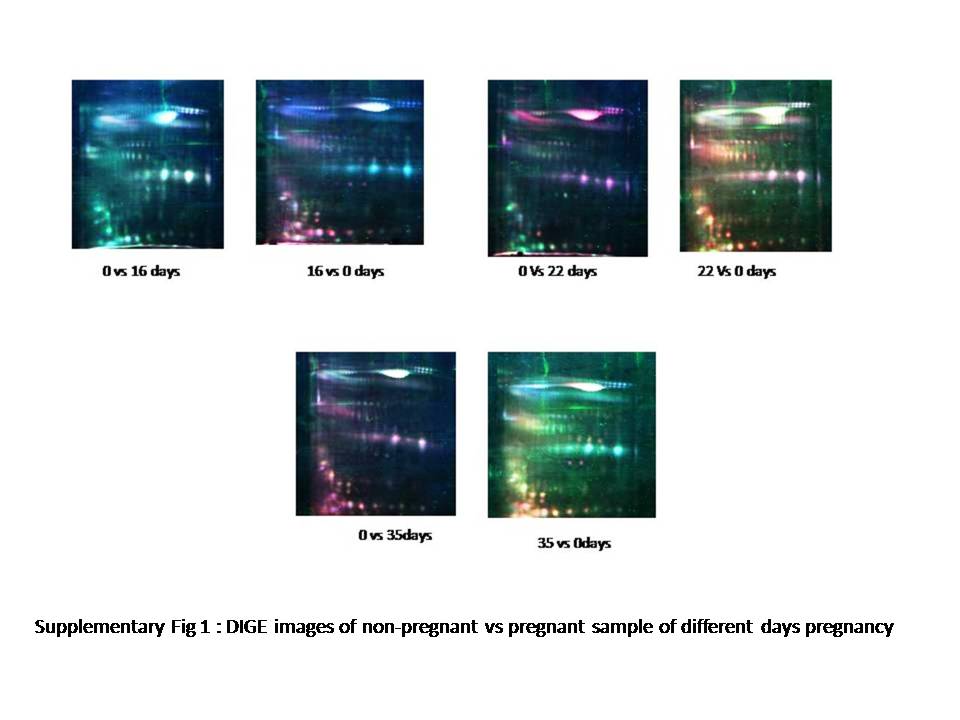

Supplement: Supplementary file 1 — 10.1186/s12014-016-9116-y Supplimentary Table: Total identified proteins revelaed by Max quant Software. [file 12014_2016_9116_MOESM1_ESM.jpg]
